# Optical Genome Mapping: A New Tool for Cytogenomic Analysis

**DOI:** 10.3390/genes16080924

**Published:** 2025-07-31

**Authors:** Brynn Levy, Rachel D. Burnside, Yassmine Akkari

**Affiliations:** 1 Department of Pathology and Cell Biology, Columbia University Irving Medical Center, New York, NY 10032, USA; 2 Department of Pathology, Immunology, and Laboratory Medicine, University of Florida, Gainesville, FL 32610, USA; rachelburnside@ufl.edu; 3 Institute of Genomic Medicine and Department of Pathology, Nationwide Children’s Hospital, The Ohio State University, Columbus, OH 43215, USA

**Keywords:** optical genome mapping, cytogenetics, cytogenomics, OGM, structural variation

## Abstract

**Background/Objectives:** Optical genome mapping (OGM) has recently emerged as a new technology in the clinical cytogenomics laboratories. This methodology has the ability to detect balanced and unbalanced structural rearrangements using ultra-high molecular weight DNA. This article discusses the uses of this new technology in both constitutional and somatic settings, its advantages as well as opportunity for improvements. **Methods:** We reviewed the medical and scientific literature for methodology and current clinical uses of OGM. **Results:** OGM is a recent addition to the methods used in cytogenomics laboratories and can detect a wide range of structural and copy number variations across a plethora of diseases. **Conclusions:** Clinical cytogenomics is an important laboratory specialty for which various technologies have been validated over the last several decades to improve detection of copy number and structural variations and their association to human disease. OGM has proven to be a powerful tool in the arsenal of clinical laboratories and provides a unified workflow for the detection of chromosomal aberrations across a wide range of diseases.

## 1. Introduction

The field of cytogenetics covers the study of chromosomes and the impact that abnormal chromosome copy number or structure has on human cell growth, function, and development. The significance of conducting chromosome studies on neoplastic specimens was recognized as early as 1914 by Boveri, who observed that solid tumors frequently exhibited highly abnormal chromosomal content [1]. It was not until 1959 that the clinical significance of constitutional chromosome abnormalities was revealed by Lejeune and colleagues, who demonstrated the presence of an extra copy of one of the smallest human chromosomes in individuals with Down syndrome [2]. Performing chromosome studies routinely for clinical purposes would have to wait another decade until simpler methods for analyzing chromosomes from peripheral blood (early 1960s) [3,4,5,6,7] coupled with banding techniques (early 1970s) [8,9,10,11,12] were developed. While most of the common aneuploidies had been described by the end of 1969, the development of chromosome banding techniques significantly enhanced the clinical diagnostic capabilities of the field of cytogenetics by enabling the additional detection of structural chromosomal changes and their correlation with clinical phenotypes [13].

Coupling molecular techniques with chromosome preparations in the 1970s and 1980s led to the development and rapid use of fluorescence in situ hybridization (FISH) probes for delineating chromosomal abnormalities that were either complex or beyond the resolution of traditional banding analysis. These probes have been widely used over the past 25 years in a clinical setting for detection of microdeletions/microduplications, chromosome enumeration in interphase nuclei and detection of specific gene-fusions resulting from chromosome rearrangements.

In the 1990s, molecular cytogenetics evolved from targeted FISH probes to genome-wide assessment with the introduction of chromosomal comparative genomic hybridization (CGH) [14]. While CGH was initially used for detection of copy number imbalances across the genome in neoplastic samples, it also demonstrated clinical utility in patients with constitutional chromosome abnormalities [15]. Chromosomal CGH transformed into array CGH (aCGH) and SNP arrays, as genomic techniques followed the same pathway as the microchip [16,17].

The first decade of the 21st century witnessed increased utilization of chromosomal microarray analysis (CMA) for improved diagnostic yields in patients with congenital abnormalities and intellectual disability [18,19]; while CMA in prenatal [20] and neoplastic [21] samples only gained momentum in the second decade of the 2000s. The next major shift in technology for chromosome analysis came with next generation sequencing (NGS) advances. NGS has been used for both targeted (e.g., NIPT) and whole genome chromosome assessment, both of which require bioinformatic resources.

Today, the term “Cytogenomics” is preferred over cytogenetics when referring to advanced molecular technologies such as microarrays or NGS to detect genome-wide chromosome abnormalities. While whole genome sequencing has the potential to uncover single nucleotide variants (SNVs), copy number variants (CNVs) and structural variants (SVs), specific and often complex algorithms, pipelines, and validations are required for revealing and reporting each of them. Whole genome sequencing (WGS) is only gradually being adopted by clinical laboratories for these purposes. High costs for purchasing, setting up and running WGS as well as the bioinformatic resources required have contributed to the slow uptake of WGS for routine clinical cytogenomic diagnostics. As such, most clinical laboratories still perform multiple targeted assays (e.g., karyotyping, FISH and microarray) and often in a tiered approach. Each assay has advantages and limitations—G-banded chromosome analysis provide single-cell resolution of mosaicism and neoplastic clones and is relatively inexpensive to perform but is hampered by relatively poor resolution. FISH is a rapid and inexpensive assay that achieves higher resolution but DNA outside of the targeted region is not interrogated at all. CMA is also a fairly rapid technique that achieves much higher whole genome resolution than G-banded chromosome analysis, but the single cell resolution for mosaicism/clonality is lost and balanced structural variants cannot be detected. NGS methods provide information at the single nucleotide level, but complex structures cannot be easily resolved. Regardless, these more established methodologies remain integral to the cytogenomics laboratory because of the advantages they provide.

A “next generation cytogenomic tool”, in the form of optical genome mapping (OGM), offers high-resolution, genome-wide detection of balanced and unbalanced structural variants and copy number information independent of cell division and has recently demonstrated excellent proficiency for detecting constitutional and somatic abnormalities [22,23,24,25,26,27,28]. In addition, OGM offers the potential for a single assay that would otherwise require karyotyping, multiple rounds of FISH testing and chromosomal microarray analysis. This review will discuss OGM technology, its increased utilization in a clinical setting and illustrate its potential advantages in terms of addressing diagnostic yield, economic impact, cytogenomic technologist workforce shortage issues, and global equity of care.

## 2. Technological Foundations of OGM

### Principles and Methodology

In the early 1990s, a technique using restriction enzyme digestion of fluorescently labeled DNA entrapped in an agarose gel matrix, with subsequent microscopic visualization of digested fragments, was pioneered in the lab of David Schwartz at NYU to map long stretches of Saccharomyces cerevisiae (*S. cerevisiae*) DNA [29]. This technique was initially termed optical mapping and laid the foundation of what we now refer to as OGM. It was developed to overcome challenges in mapping genes and chromosomes of prokaryotes and simple eukaryotes prior to the completion of the Human Genome Project and invention of next generation massively parallel sequencing methods (NGS). With large gaps in reference genomes due to inherent limitations of sequencing technology and inability to span repetitive or other complex elements, optical mapping overcame some of these technical challenges by using ultra-high molecular weight DNA. These very long fragments spanned many of the troublesome regions and served as a scaffolding reference for aligning sequencing contigs. In 2008, Kidd et al. published the first use of optical mapping on human genomes to interrogate structural variation among phenotypically normal individuals using a similar alignment method [30]. The group showed the vast structural variation not previously appreciated using short-read sequencing methods [30].

**Figure 1:**
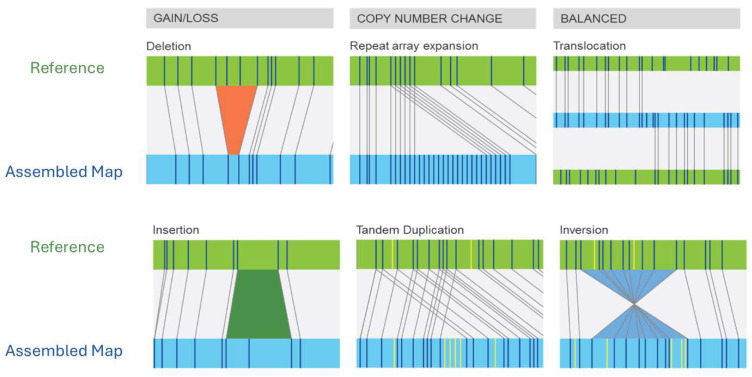
Illustration of the spectrum of chromosomal aberrations detected by OGM. Each diagram shows the reference genome (green) compared to a patient genome (blue). Bioinformatic alignment of patient molecules against the reference can reveal altered labeling patterns consistent with structural variation. © Copyright, 2025, Bionano Genomics, Inc.

Unlike early methods that labeled genomic DNA prior to enzymatic digestion, modern methods use fluorescent labeling of restriction enzyme motifs themselves (without enzyme digestion), averaging one labeled motif every 6–15 kb in the human genome [31]. Moreover, instead of fixing linearized DNA in an agarose gel matrix, optical mapping linearizes genomic DNA through progressively narrower channels (micro- to nanochannels), with laser imaging capturing label patterns as the DNA passed through the nanochannels. Bioinformatic algorithms are then used to align molecules to a reference genome with known labeling patterns. A loss (deletion) of chromosomal material will be evident as missing label patterns in the patient compared to the reference while a gain of chromosomal material will be revealed by regions with extra label patterns (Figure 1). Extra label patterns can further be identified as insertions or repeat expansions (Figure 1). Balanced rearrangements, such as translocations and inversions, will be identified when the label patterns in a single patient DNA molecule first map to one chromosomal region and then contiguously map to another chromosomal region (Figure 1). The DNA fragments generated by OGM are typically around 150–300 Kb and using the reference genome, gene-level information can be gleaned in aberrant regions. As such, cytogeneticists may consider optical mapping akin to an ultra-extended G-banded karyotype with a thousand-fold increase in resolution. The resolution attained by OGM analysis depends on the analysis pipeline employed with the rare-variant analysis pipeline typically used for surveying neoplastic DNA and the *de novo* assembly pipeline used for analyzing germline DNA. Specific details of the data collected and resolution of each pipeline are shown in Table 1 and comparison of OGM features compared to other genomic analysis techniques is shown in Table 2.

## 3. Clinical Applications of OGM

### 3.1. Hematologic Malignancies and Solid Tumors

Hematologic malignancies and solid tumors are largely driven by hallmark chromosome abnormalities with diagnosis, prognosis and clinical management guided by professional organizations such as the World Health Organization (WHO), the National Comprehensive Cancer Network (NCCN), the European Leukemia Network (ELN), and the Children’s Oncology Group (COG). Detection of these abnormalities by routine cytogenomic methods typically requires both G-banding analysis and extended FISH panels to capture relevant aberrations. Atypical abnormalities not covered by FISH panels will escape detection. The success of traditional methods is challenged by culture failures, insufficient metaphase cells, poor banding and limited FISH probe sets [32]. G-band analysis also relies on technical expertise for subjective identification of abnormalities which has only become more challenging given the current workforce shortage in clinical cytogenetic laboratories [32,33]. Optical genome mapping offers an unbiased assessment of the entire genome without the requirement for *a priori* selection of targeted loci/regions. This has proven to be particularly effective for uncovering novel gene-fusions, gene-fusion partners, and cryptic copy number changes [34,35,36,37]. The ability to directly examine DNA from uncultured cells eliminates artifacts secondary to selective culture growth advantages as well as abnormal clones failing to propagate in culture. A distinct advantage of OGM in hematologic malignancies is the ease of extracting high molecular weight DNA from bone marrow and/or neoplastic blood specimens.

Recent studies using OGM for the assessment of hematologic malignancies have shown improved diagnostic yields, risk stratification, and patient eligibility for clinical trials [24,38]. For example, a 2023 multicenter study on patients with acute myeloid leukemia (AML) identified clinically relevant SVs/CNVs in 13% of cases that had been missed by the routine methods, uncovered results that would alter recommended clinical care in 4% of cases, and revealed findings that would render patients eligible for clinical trials in 8% of cases [24]. With the explosion of clinical studies using OGM in patients with hematologic neoplasms, an international consortium was formed to provide guidance for laboratories implementing OGM as a standard-of-care cytogenomic assay for the diagnostic workflow in various clinical settings [23,25]. The framework and recommendations detailed by the consortium also covered critical laboratory issues such as validation, quality control and analysis and interpretation of variants [23,25].

OGM studies in patients with solid tumors have similarly demonstrated increased diagnostic yields with ensuing changes in clinical management. Examples include gliomas [39], neuroblastoma [40], and prostate cancer [41]. Even though OGM cannot be performed with low molecular weight DNA extracted from FFPE samples, snap frozen or fresh specimens derived from any solid tumors are highly amenable to testing by OGM. Indeed, several studies have provided guidance regarding pre-analytic and analytic parameters to increase the success of OGM in solid tumors [42,43].

### 3.2. Constitutional Studies

Similar to the published value of OGM in oncology, a parallel set of data has been reported in constitutional genomics. For pediatric cases, OGM was assessed by several groups comparing standard of care methodologies in patients referred for routine cytogenomic testing [22,44,45]. Structural and copy number variants, as well as regions of homozygosity were detected by OGM and classified according to current guidelines. These reports demonstrated high concordance with other methodologies and provided valuable suggestions for process improvement and filtering strategies. A particular strength of OGM is its ability to resolve complex rearrangements as well as uncover cryptic balanced rearrangements in patients with phenotypes [34,46,47,48,49]. While CMA can identify the imbalances involved in complex rearrangements, it fails to delineate the architecture of unbalanced rearrangements and is unable to identify balanced events like translocations, a strength attributed to OGM. Furthermore, the unique ability of OGM to screen for repeat expansion and contraction disorders like Fragile X and Facioscapulohumeral muscular dystrophy (FSHD) was highlighted by Iqbal and colleagues and has now been demonstrated for various other repeat expansion disorders such as Friedreich’s Ataxia, Progressive Myoclonic Epilepsy 1A, Fuchs endothelial corneal dystrophy, spinocerebellar ataxia, myotonic dystrophy types 1 and 2, and cerebellar ataxia, neuropathy, and vestibular areflexia syndrome (CANVAS) [22,50,51,52,53].

In the prenatal setting, several groups reported on the emergence of OGM as an effective testing tool for all standard prenatal indications, including: follow up for abnormal non-invasive cell-free DNA test or serum screen, family history with suspicion of a chromosomal abnormality, and structural ultrasound abnormalities [26,54,55,56]. Prenatal OGM studies have demonstrated high accuracy compared to standard of care testing with high positive predictive value and concordance [26,54,55,56]. These studies have also underscored the value of employing a more comprehensive and sensitive technique, thereby reducing or even eliminating the need for tiered testing. While screening for expansion/contraction disorders has never been within the scope of traditional cytogenomic prenatal testing, its potential inclusion was explored and discussed by Levy et al. as part of their multicenter prenatal OGM study [26].

Another important application of OGM is in the field of infertility. Conventionally, couples with multiple miscarriages are often referred for standard G-banded chromosome analysis to rule out a balanced structural rearrangement. In these cases, OGM can offer a higher resolution method to identify these rearrangements and can provide valuable information regarding gene content and more precise breakpoints [57].

## 4. Advantages of OGM

### 4.1. Resolution of Complex Genomic Rearrangements

The ultra-high molecular weight DNA molecules assessed by OGM provides an opportunity to resolve the genomic architecture of complex SVs with a high degree of accuracy [58,59,60,61,62]. These long molecules tend to span both sides of an SV and can reveal fusion breakpoints following bioinformatic alignment to reference chromosomes. In contrast, CMA only reveals CNVs associated with complex SVs but cannot discern the orientation and complex architecture of those SVs nor can it detect balanced (copy neutral) SVs.

### 4.2. Analysis of Repetitive Regions and Tandem Repeats

Resolving CNVs and SVs in complex regions with repetitive elements, including segmental duplications and centromeric/telomeric repeats, remains a limitation for most standard methodologies, including OGM [63]. The clinical impact of these repetitive elements varies. For example, segmental duplications have no inherent pathogenic effect but rather play a key role in facilitating the formation of pathogenic CNVs through non-allelic homologous recombination events. In contrast, other repetitive elements, like repeat expansions and contractions, are directly associated with specific genetic disorders such as Fragile X syndrome and Facio-Scapulo-Humoral dystrophy (FSHD). Innovation in the OGM bioinformatic pipeline [52] has facilitated the detection of numerous trinucleotide pathogenic expansions as well as a contraction of the number of D4Z4 repeats associated with FSHD [22,50,51,52,53,64,65,66,67]. Large polynucleotide repeat expansions may also be detected and have now been demonstrated for CANVAS, amyotrophic lateral sclerosis/frontotemporal dementia syndrome, spinocerebellar ataxia type 10, myotonic dystrophy type 2, spinocerebellar ataxia type 36, and familial adult myoclonic epilepsy type 2 [52,68]. Many of the neuromuscular disorders associated with expansion disorders are linked to a multitude of different genes/expansion motifs, and comprehensive clinical testing for patients affected with these disorders has proven challenging. Given the complexity of the genomic architecture of these repetitive elements, a single assay that can screen the whole genome for expansion disorders represents a significant technological advance over current gold standard methods. For each of these disorders, laboratories will need to establish the sensitivity of OGM to detect expansions at the border of premutation and expansion and should have clear reporting criteria for cases that fall in the gray zone [22,69].

### 4.3. Time and Cost-Effectiveness in Clinical Settings

Several international laboratories have adopted OGM as a primary cytogenomic methodology in preference to the combination of chromosome analysis, FISH, and CMA [23,70,71]. As the entire OGM assay can be completed in less than one week, it is a feasible single-assay option for resource-constrained laboratories who are looking for a comprehensive genomic technology that would provide maximum diagnostic ability without the requirement for simultaneous or cascade testing [33,72,73]. For laboratories that already offer testing by multiple technologies, OGM offers the ability to shorten the time to diagnosis by minimizing the number of successive tests required as well as reducing overall costs.

## 5. Limitations of OGM

### 5.1. Technical Limitations and Areas for Improvement

While OGM has been proposed as a total replacement technology for traditional cytogenomic methods such as G-banded chromosome analysis, FISH, and CMA [74], recognizing its distinct limitations is important when laboratories consider validating it for clinical use.

The DNA fragment length required for OGM is considerably longer than that obtained using routine molecular testing such as PCR, CMA, and sequencing. As such, most archival DNA specimens will not be amenable to OGM. Archival tissue specimens, if stored appropriately, may provide an adequate DNA source for OGM when using a suitable DNA extraction methodology for ultra-high molecular weight DNA (UHMW). The requirement for UHMW DNA limits the types of tissues that can be used and currently eliminates the possibility of studying formalin-fixed paraffin embedded (FFPE) tissues. The recommended minimum number of cells required to yield sufficient DNA is ~1 M cells. Certain sample types, such as amniotic fluid may not always have the required amount of cells, which would necessitate cell culturing and consequently extend the turnaround time for reporting. In the prenatal context, this is no different from the CMA workflow, which reflexes to cell culturing when a very small cell pellet is observed in amniotic fluid. The 1 million cell recommendation has been set by the manufacturers, and some investigators have been successful in validating OGM with as little as 400,000 cells [55].

Detecting long contiguous stretches of homozygosity (LCSH; also known as AOH/ROH/LOH) indicative of either uniparental disomy (UPD) or consanguinity is readily achievable by most CMA platforms that integrate SNP probes. The size resolution for LCSH detection by CMA depends on the number and spacing of SNP probes but can be as small as 1 Mb and is often reported when ≥5 Mb [75]. The detection of LCSH by OGM is currently limited to a minimum of 20 Mb, which may miss certain cases of UPD, with smaller LCSH regions [75]. Improvements in the OGM algorithm for the detection of smaller LCSH regions are expected over time.

Similar to CMA and NGS, OGM assesses aggregated DNA from a population of cells. As such, single cell-level analysis is not possible. This has implications for cancer studies where clonal heterogeneity is often detected by more conventional methods of chromosome analysis and FISH. However, it can be argued that since OGM analyzes single molecules coming from around 1 million cells, the clonal fraction estimated by OGM is more resolute than the standard analysis of twenty G-banded metaphases [24,42,76,77]. In addition, as OGM does not require cell culture, the actual subclone frequency may be more accurately reflected and not biased by cultural artifacts [24]. OGM certainly does not have the power of single cell targeted FISH in the context of residual disease.

### 5.2. Data Interpretation and Variant Classification

While OGM data interpretation is similar to CMA for CNVs, there are some differences that users should be aware of during analysis. For example, insertions—which may be duplications or even polynucleotide repeat expansions—will often require orthogonal testing to confirm. Small insertions flagged by OGM may contain an insufficient number of labels, preventing their unambiguous identification and endogenous location. Most of these small insertions fall well below most laboratories’ threshold for reporting or are in gene-poor regions. As several repeat expansion disorders are associated with repeats localized to non-coding regions of genes, laboratories may wish to highlight such regions in a user-defined bed file during analysis to ensure that these regions are not overlooked.

OGM’s capacity to resolve complex SVs with a high degree of accuracy may potentially pose turnaround time challenges to clinical laboratories as they wade through a new data format and sift out pertinent clinical information from the plethora of data. As with CMA, it may be possible to combine complex events under a more generalized ISCN nomenclature, using the “cx”, “cha”, or other abbreviations outlined in the ISCN [78], but CNVs or SVs that have diagnostic, prognostic, or therapeutic implications will still need to be reported out separately. The 2024 edition of the ISCN introduced OGM nomenclature rules [78].

Detection of SVs is facilitated by alignment of molecule labeling patterns of the patient DNA against those of a reference genome (Figure 1). With respect to breakpoint resolution, SV breakpoints most often occur between two labels, which the analysis software may call as a range of genomic coordinates between the flanking labels for each SV breakpoint. This is similar to CMA, which calls CNV breakpoints at a genomic coordinate corresponding to the first non-2n probe [79]. The ISCN does provide examples using the karyotype long version format for reference.

### 5.3. Integration into Existing Clinical Workflows

As with any new technology, open communication between the laboratory and the clinical provider regarding implementation of a new technology is highly recommended. Such interaction could educate the healthcare provider on the advantages and limitations of OGM, thus facilitating appropriate referrals and patient testing. For most patients, an algorithm testing approach utilizing all available testing modalities is dictated by the specific disease and clinical phenotype of the patient, as well as payer coverage of assays. In this sense, OGM results could inform or clarify previous results of conventional methods or even be used as a primary method, all of which depend on resources and cost-effectiveness of implementation. As advocacy efforts for the creation of an OGM CPT code continue to be successful, it is anticipated that OGM will be more widely adopted in clinical genomic laboratories.

## 6. Concluding Remarks

Since the first proof of principle studies appeared showing the technical capabilities of OGM, there has been an exponential increase in the number of manuscripts in the literature demonstrating the clinical utility and validity of OGM across multiple specimen types and diseases. The multi-center nature of many of these investigations has further established OGM as a *bona fide* testing modality in the clinical laboratory. This technology can be seen as a single assay that encompasses the power of G-banding, FISH and CMA with an added benefit of screening for repeat expansion/contraction disorders, bearing in mind its current technical limitations. With limited healthcare resources and increasing shortages in the laboratory workforce, OGM is ideally suited to offset some of these challenges.

## Figures and Tables

**Table 1:** Metrics of OGM sensitivity and resolution across various interpretation pipelines and chromosomal aberrations.

Application	Germline DNA Analysis	Somatic DNA Analysis
Data collected	400 Gbp	1.5 Tbp
Coverage setting	100×	400×
Effective coverage	80×	300×
Variant allele frequency	≥50%	≥5%
Analysis pipeline	*De Novo* Assembly	
		
**Resolution by variant type at >90% sensitivity**		
Insertions	>500 bp	>5 kbp
Deletions	>700 bp	>7 kbp
Repeat expansion/contractions	>500 bp	>5 kbp
Duplications	>30 kbp	>150 kbp
Translocations	>70 kbp	>70 kbp
Inversions	>30 kbp	>70 kbp

**Table 2:** Comparison of features of genomic methods. FISH; fluorescence in situ hybridization, CMA; chromosomal microarray, GS/ES; genome sequencing/exome sequencing, OGM; optical genome mapping. Green check = feature is available, red X = feature is not available, blue +/− = feature may be available in some contexts.

	G-Banded Chromosome Analysis	FISH	CMA	GS/ES	OGM
**Whole genome coverage**	**√**	**X**	**√**	**+/−**	**√**
**AOH**	**X**	**X**	**√**	**+/−**	**√**
**Repeat Expansions/Contractions**	**X**	**X**	**X**	**+/−**	**√**
**Resolution**	5–10 Mb	60 Kb	25 Kb	SNV	500 bp
**Limit of Detection**	Single Cell	Single Cell	~10%	~1–5%	~20%
**Detects Balanced SVs**	**+/−**	**+/−**	**X**	**√**	**√**
**Bioinformatics required**	No	No	No	Yes	Yes
**Turnaround Time**	5–28 days	24 h–5 days	~7 days	~4 weeks	~7 days
**Cost**	$	$	$$	$$$$	$$
